# Clonal reproduction as a driver of liana proliferation following large‐scale disturbances in temperate forests

**DOI:** 10.1002/ajb2.70085

**Published:** 2025-08-13

**Authors:** Hideki Mori, Takashi Kamijo

**Affiliations:** ^1^ Forestry and Forest Products Research Institute Matsunosato 1, Tsukuba Ibaraki 305‐8687 Japan; ^2^ Faculty of Life and Environmental Sciences University of Tsukuba 1‐1‐1 Tennodai, Tsukuba Ibaraki 305‐8572 Japan

**Keywords:** Apocynaceae, clonal growth, clonal proliferation, forest disturbance, forest succession, *Trachelospermum asiaticum* var. *asiaticum*, vegetative reproduction, volcanic eruption, woody vine

## Abstract

**Premise:**

Large‐scale disturbances significantly impact forest dynamics, structure, and biodiversity. Lianas proliferate rapidly after such events, likely through clonal reproduction. Understanding this process is challenging because it requires precise disturbance history and accurate estimation of whether individuals originated from clonal reproduction, seed reproduction, or preexisting vegetation.

**Methods:**

We examined whether clonal reproduction drives liana proliferation in both early successional and mature forest conditions by analyzing the dominant liana species (*Trachelospermum asiaticum* var. *asiaticum*; Apocynaceae) in temperate forests on a volcanic island. The study included young forests recovering from a volcanic eruption 22 yr ago and old‐growth forests unaffected by eruptions for >800 yr. We established six 100 m^2^ quadrats (three in each forest type), sampled 586 individuals, and used 11 microsatellite markers to assess genetic structure.

**Results:**

Significant clonal expansion was observed in both forest types, but stem density and genetic diversity varied markedly. Old‐growth forests had 14 times greater stem density and five times more genets (clones) than young forests, and exhibited unexpectedly greater clonal diversity despite their advanced successional stage. This indicates that clonal reproduction results in high abundance in both forest conditions, while both seed and clonal reproduction enhance clonal diversity in old‐growth forests.

**Conclusions:**

Our analysis revealed that a few genets, recruited via seed dispersal in early successional stages, rapidly expanded through extensive clonal reproduction, leading to long‐term liana proliferation. These findings highlight how clonality and seed recruitment, together with environmental changes during succession, shape the population dynamics and clonal diversity of lianas following disturbances.

Large‐scale disturbances significantly alter the structure, dynamics, and functions of forests (Oliver, 1980; Seidl et al., 2014). With climate change and forest fragmentation, these disturbances are increasing globally (Laurance, 2004; Siedl et al., 2017). Evaluating the impact of anticipated long‐term increases in large‐scale disturbances on forests has become a major focus in forest research (Schurman et al., 2018; McDowell et al., 2020; Patacca et al., 2023). For instance, large‐scale wind disturbances and subsequent pest outbreaks have been shown to significantly restructure forest landscapes, altering canopy composition and facilitating species shifts (Schurman et al., 2018).

Lianas are crucial components of forest ecosystems and proliferate rapidly following such disturbances (Ladwig and Meiners, 2015). Typhoons, wildfires, forest fragmentation, and other events can trigger long‐term increases in liana abundance worldwide (Allen et al., 2007; Leicht‐Young et al., 2013; Ngute et al., 2024). However, the biological mechanisms of post‐disturbance liana proliferation are poorly understood. The ability of lianas to quickly colonize and dominate disturbed areas often increases competition with tree species, potentially altering forest regeneration and ecosystem dynamics (Schnitzer and Bongers, 2002).

Lianas establish and colonize new environments after disturbances through seed dispersal, preexisting plant propagules (including existing seeds and seedlings), and clonal reproduction (Schnitzer and Bongers, 2002). The results of previous studies indicated that clonal reproduction is a major driving factor for their long‐term proliferation (Yorke et al., 2013; Schnitzer et al., 2021). For instance, the increase in lianas over a decade following small‐scale natural disturbances has been largely attributed to stem resprouting (Schnitzer et al., 2021). Clonal reproduction in lianas has been documented in several studies (Putz, 1984; Sakai et al., 2002; Schnitzer et al., 2012; Mori et al., 2021), highlighting its significant role in their abundance and spatial distribution patterns in forests.

The extensive clonal reproduction of plants by stolons or rhizomes not only increases their abundance but also alters their genetic diversity, including genetic structure and clonal diversity, depending on the growth strategies employed (Schmid and Harper 1985). The “guerilla” type establishes low‐density ramets over a large area, which subsequently leads to high density through intermingling of clones. Conversely, the “phalanx” type establishes and maintains high‐density ramets in a small area, exhibiting a lack of intermingling and strong exclusion of other plants. Additionally, intermingling of clones has been demonstrated to enhance overall fitness in clonal plants (Matsuo et al., 2014). Clonal diversity is often observed to decline over time as a result of clonal expansion by a few surviving genets. For example, in dwarf bamboo, high initial clonal diversity following mass flowering tends to diminish as dominant genets spread clonally (Makita, 1992; Kitamura and Kawahara, 2009). Therefore, an understanding of these growth types and the degree of intermingling among clones, together with their consequences for clonal diversity over time, is essential for comprehending the demography and survival strategies of clonal plants, including lianas.

Despite the importance of clonal reproduction for liana proliferation, its mechanisms following large‐scale disturbances are not fully understood. This is likely due to the need for precise disturbance history and the challenge of evaluating clonal reproduction alone, as established individuals may originate from clonal reproduction, seed reproduction, or preexisting vegetation. Notably, under less severe disturbances, clonal plants can rapidly regenerate by sprouting from surviving tissues, thereby contributing to post‐disturbance recovery (Fahrig et al., 1994; Winkler and Fischer, 2001). Most existing knowledge is focused on disturbances such as forest fragmentation, typhoons, and wildfires (Allen et al., 1997; Leicht‐Young et al., 2013; Barry et al., 2015; Ngute et al., 2024), in which cases regenerative plant propagules (e.g., soil seed banks) might not be completely eradicated. Consequently, even with a clear disturbance history, distinguishing between increases in abundance from individuals that survived the disturbance and from those newly recruited after disturbance is challenging. By contrast, volcanic eruptions, particularly those characterized by thick ash deposits, can eliminate regenerative plant propagules (Antos and Zobel, 1986; Tsuyuzaki, 1987). In such instances, the increase in abundance would be driven by propagules (e.g., seeds) introduced from external sources, making it possible to accurately estimate the origins of individual plants. Further research is necessary to unravel these complex interactions and develop a comprehensive understanding of the biological mechanisms utilized by lianas following large‐scale disturbances.

In order to investigate whether the proliferation of liana species after large‐scale natural disturbances is driven by clonal reproduction, we conducted a comparative analysis of liana abundance and genetic structure in temperate forests on a volcanic island. The volcanic island was subject to a significant volcanic eruption in 2000, which resulted in the complete loss of a substantial portion of its vegetation (Kamijo et al., 2008; Kawagoe et al., 2011) (Figure 1; Appendix S1). The study sites included (1) young forests (shrublands) that were established after the complete loss of vegetation caused by the 2000 eruption and (2) old‐growth forests (estimated to be >800 yr old) unaffected by eruptions. A preliminary survey of vegetative changes in these two forest types indicated that lianas have recently colonized the young forests and remain consistently dominant in the old‐growth forests, with spatially continuous high‐density ramets on the forest floor. These distinct forest types are ideal for accurately estimating clonal reproduction in lianas by excluding the influence of preexisting plant propagules. Specifically, we asked two questions: (1) Does clonal reproduction primarily drive the initial colonization of liana species in young forests formed after a volcanic eruption? We hypothesized that, following the volcanic eruption, a few genets established via seed dispersal from surrounding areas, and that the majority of liana individuals in these young forests originated subsequently from clonal reproduction by those genets, due to the absence of regenerative plant propagules following the volcanic eruption. (2) How does clonal reproduction contribute to the long‐term increase in abundance and genetic complexity of liana species in old‐growth forests? We hypothesized that in old‐growth forests, established individuals continue clonal growth, resulting in greater stem density and less clonal diversity than in young forests, while the intermingling of these clones can produce a more complex spatial genetic structure.

**Figure 1:**
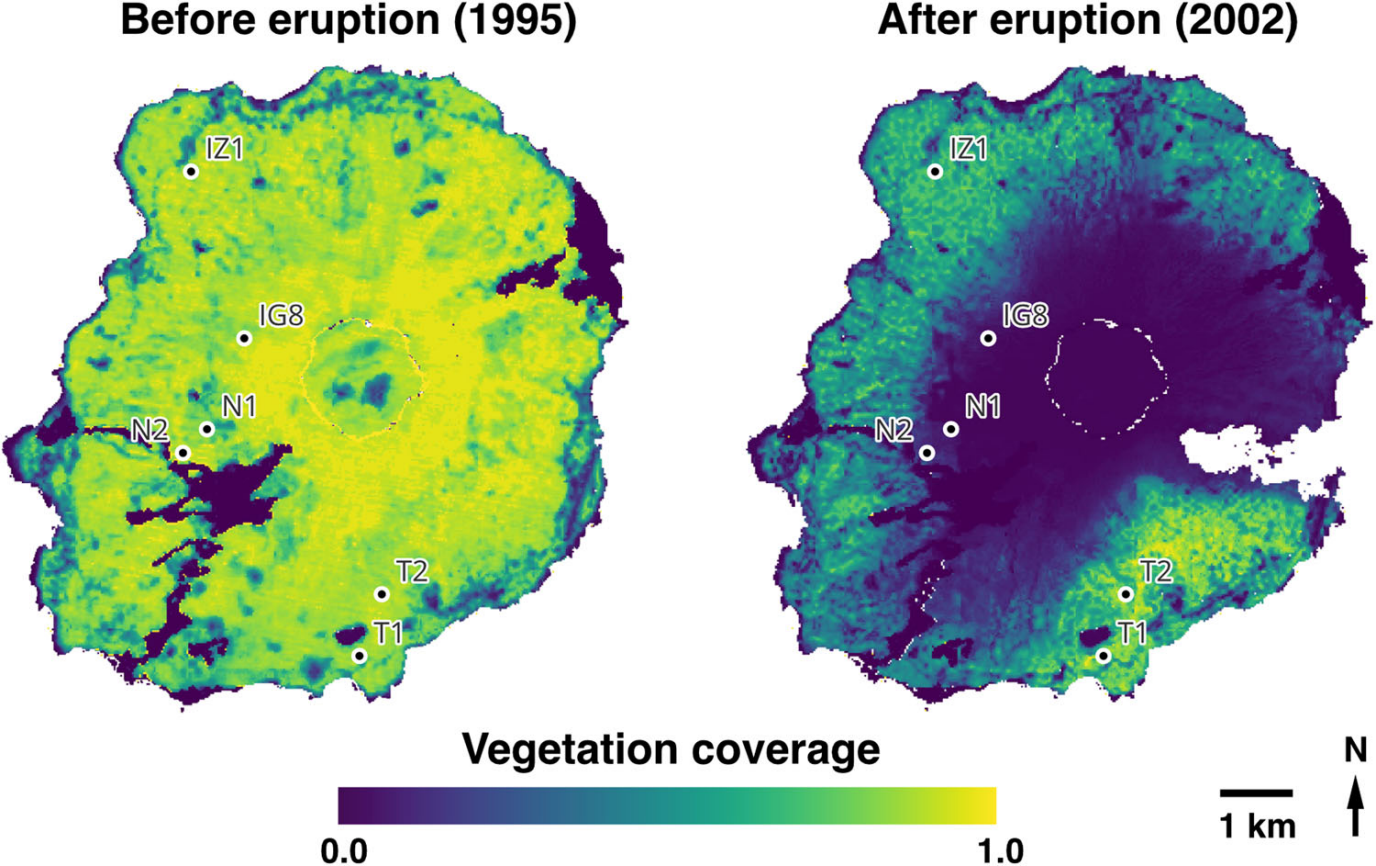
Study‐site locations and vegetation coverage on Miyake‐jima Island before and after the 2000 volcanic eruption. Maps based on Yamanishi et al. (2003).

## MATERIALS AND METHODS

### Study sites

The study was conducted on Miyake‐jima Island, Tokyo prefecture, Japan (Figure 1). Miyake‐jima Island experienced a significant volcanic eruption in 2000, resulting in substantial ecological transformations (Yamanishi et al., 2003; Takahashi et al., 2008; Katoh et al., 2020; Peng et al., 2021). The eruption resulted in the complete loss of vegetation and creation of bare land on large parts of the island (Kamijo and Hashiba, 2003; Kamijo et al., 2008; Kawagoe et al., 2011; Figure 1; Appendix S1). Previous research on primary succession and vegetative changes in Miyake‐jima Island found that the colonization of *Alnus sieboldiana* (Betulaceae) begins after 16 yr of primary succession, forming an *Alnus* shrubland after 37 yr of primary succession; and the colonization of *Castanopsis sieboldii* (Fagaceae) occurs after >800 yr of primary succession (Kamijo et al., 2002; Appendix S2). The present study utilized long‐term vegetation monitoring sites (Kamijo et al., 2002; Peng et al., 2021; Appendix S3) to select two distinct forest communities for use in analyzing the clonal structures of lianas that have developed with and without large natural disturbances. The first forest type is an evergreen broadleaf forest dominated by *C. sieboldii* that has remained unaffected by volcanic activity for >800 yr (hereafter “old‐growth forest”). The second type is a deciduous shrubland dominated by *A. sieboldiana*, currently in the recovery phase following the destruction of vegetation due to the 2000 eruption (hereafter “young forest”).

The old‐growth forests are characterized by greater canopy height and vegetative coverage in comparison to the young forests, indicating less light availability in old‐growth forests (Appendix S4). Among the young forest sites, IG8, which is located closest to the crater, had less canopy cover than N1 and N2 in 2018, suggesting relatively greater light availability at that time (Appendix S4), likely due to delayed vegetation recovery in IG8 caused by its proximity to the crater. However, by 2022, the canopy at IG8 had largely closed, indicating that differences in light availability among the young forest quadrats were likely less pronounced at the time of our study. In addition, the two forest types differ in soil composition: the old‐growth forests are classified as Andosols (Allophanic Andosols), while the young forests are classified as Regosols (Volcanogenous Regosols) (NARO, Japanese Soil Inventory, 2025). A key distinction between these soil types is the presence of volcanic ash, with no ash deposits in the old‐growth forests, whereas the young forests are characterized by a thick volcanic ash layer (23–38 cm; Appendix S5). Volcanic ash‐rich soils typically have low nitrogen availability, reflected in high C/N ratios. At one young forest site (IG8), the C/N ratio in 2007, 2011, and 2019 was reported to be 6.82–24.52, 5.46–18.14, and 3.87–15.78, respectively, showing a significant decline over time, likely influenced by nitrogen fixation by *A. sieboldiana* (Peng et al., 2021).

### Study species

The study species is *Trachelospermum asiaticum* (Siebold et Zucc.) Nakai var. *asiaticum* (Apocynaceae), with its nomenclature based on Yonekura and Kajita (2003). *Trachelospermum asiaticum* var. *asiaticum* is a woody climbing plant native to warm temperate forests, classified as a wind‐dispersed, evergreen broadleaf species. The climbing type of this species is classified as a root climber (Kusakabe et al., 2023), utilizing adhesive roots to attach to and ascend host trees, though it occasionally exhibits twining behavior around supports. On Miyake‐jima Island, *T. asiaticum* var. *asiaticum* is a dominant species in the forest understory and is found across a range of forest types, from young forests to old‐growth forests (Appendices S1 and S2). It is particularly abundant in old‐growth forests, forming dense, carpet‐like vegetation that spreads across the forest floor, creating apparent individuals (ramets) while forming genetically identical clones (genets). The results of previous vegetation surveys in young and old‐growth forests indicated that this species had established and colonized after the large natural disturbance (Appendix S3). Given its remarkable clonal growth characteristics in a range of forest types, *T. asiaticum* var. *asiaticum* is an ideal species for achieving the objectives of this study.

### Sampling and DNA extraction

Sampling was conducted at long‐term monitoring sites located within young and old‐growth forests on Miyake‐jima Island in 2022 (Figure 1). In each forest type, three quadrats (10 × 10 m) were selected for sampling (young forest sites: N1, N2, IG8; old‐growth forest sites: T1, T2, IZ1; Figure 1), resulting in a total of six 100 m^2^ quadrats. Each quadrat was divided into 1 × 1 m grids (totaling 100 grids per quadrat), and a ramet was randomly sampled within each grid. Lianas are known to exhibit two distinct stages of clonal growth: the on‐floor stage, characterized by small individuals before they begin climbing trees, and the on‐tree stage, during which individuals ascend trees (Mori et al., 2021). Therefore, sampling was conducted for both life stages, resulting in a potential maximum of 200 samples per quadrat. Following collection, samples were placed in paper bags with silica gel to maintain dryness and stored at room temperature until DNA extraction. DNA was extracted using the 2 × CTAB (cetyltrimethylammonium bromide) protocol (Murray and Thompson, 1980).

### DNA marker development and genotyping

Polymerase chain reaction (PCR) was performed using 11 microsatellite markers designed for the study species (Appendices S6 and S7). Sequences of *T. asiaticum* var. *asiaticum* were downloaded from the Sequence Read Archive (accession no. SRR6425626) at NCBI (Bethesda, Maryland, USA; http://www.ncbi.nlm.nih.gov/sra), and 11 loci of microsatellite markers (Appendix S6) were developed using the CMIB (CD‐HIT‐EST, MISA, ipcress, and BlastCLUST) pipeline (Ueno et al., 2012), following the methodology described by Mori et al. (2016; for details of marker development, see Appendix S7). The PCR products were analyzed using a 3130 Genetic Analyzer (Applied Biosystems, Foster City, California, USA). Electropherograms from each marker were meticulously examined for peak patterns using GeneMarker version 1.95 (SoftGenetics, State College, Pennsylvania, USA).

### Data analysis

Clones were identified using methods proposed by Arnaud‐Haond et al. (2007). First, the ability of the microsatellite markers to distinguish multilocus genotypes (MLGs) was tested by calculating the number of MLGs for all combinations of a given locus, and then the results were verified based on the plateaus of the genotype accumulation curves (Appendix S8). To ascertain whether stems of the same MLG belonged to the same clone, the probability of a given MLG occurring in a population under Hardy‐Weinberg equilibrium (*P*_gen_) was calculated using the equation $P_{\text{gen}}=\sum_{i=1}^{l}(f_{i})2^{h}$ (Parks and Werth 1993), where $f_{i}$ is the frequency of each allele at the *i*th locus estimated with a round‐robin method, and *h* is the number of heterozygous loci. Then, the probability of obtaining *n* repeated MLGs from a population more than once by chance in *N* samples (*P*_sex_) was calculated using the equation $P_{\text{sex}}=\sum_{i=n}^{N}\frac{N!}{i!(N-i!)}{\left[P_{\text{gen}}\right]}^{i}{\left[1-P_{\text{gen}}\right]}^{N-i}$ (Parks and Werth, 1993). To distinguish each distinct MLG that belonged to a distinct clone, multilocus lineages (MLLs) were defined based on pairwise genetic distances. This procedure was necessary to prevent the false detection of clones due to slightly different MLGs resulting from somatic mutation or genotyping errors.

The pairwise genetic distance threshold was determined with the “cutoff_predictor” function of R package “poppr” (Kamvar et al., 2014, 2015). MLLs are equivalent to genets (clones) in ecological studies, and we will use “genets” hereafter for MLLs. Finally, to evaluate the clonal diversity the following indices were obtained: clonal richness (*R*), Simpson's evenness index (*V*), and the Pareto index (*β*) (Arnaud‐Haond et al., 2007). Clonal richness (genotypic richness) was calculated as R=(G−1)/(N−1) where *G* is the number of genets in *N* samples. Simpson's evenness index was calculated as $V=(D-D_{\min})/(D_{\max}-D_{\min})$ where *D* is the Simpson index $D=1-\sum_{i=1}^{G}\left[n_{i}(n_{i}-1)/N(N-1)\right]$, $D_{\min}=\{[\left(2N-G\right)\left(G-1\right)]/N^{2}\}\times [N/(N-1)]$, $D_{\max}=[(G-1)/G]\times [N/(N-1)]$, *G* is the number of unique MLGs, and *N* is the number of samples. The Pareto index is the negative value of the slope of the power law (Pareto) distribution of clonal membership. The equation for Pareto index is $N_{\geq X}=\alpha X^{-\beta }$ where $N_{\geq X}$ is the number of genets containing *X* or more ramets. The Pareto index (*β*) is higher when genets have a greater density and the distribution of ramets per genet has greater evenness. Calculation of *P*_gen_, *P*_sex_, and the clonal diversity indices were conducted using the R package “RClone” version 1.0.3 (Bailleul et al., 2016).

Differences in liana abundance and in the distribution of genets between the study sites were examined in terms of stem density, genet size, and number of overlaps between genets. Stem density in the six quadrats was estimated using ten 1 × 1 m sub‐quadrats randomly established within a 10 × 10 m quadrat, resulting in a total of 60 sub‐quadrats in the six 100 m^2^ quadrats. The total number of stems (ramets) within ten 1 m^2^ sub‐quadrats were counted for each 100 m^2^ quadrat. The size of each genet was defined as the minimum convex polygon (convex hull) enclosing the genet. The calculation of the genet size was performed using QGIS version 3.30 (QGIS Development Team, 2024). Differences in stem density and genet size among the six quadrats were tested with Tukey's multiple comparison test using the R package “emmeans” (Russell, 2024). To test whether the total number of overlaps among genets differed among the six quadrats, the jack‐knife procedure described in Mori et al. (2018) was used, with the mean and 95% confidence intervals (CIs) for each quadrat being obtained from data sets generated by excluding one genet. This was done for all combinations of genets. If the contribution of a single genet to the total number of genet overlaps was significant, then the 95% CI would be high in comparison to that of the mean value. We considered the values between the quadrats to be significantly different when their 95% CIs did not overlap. The data analysis was done using R version 4.3.2 (R Core Team, 2023).

## RESULTS

### Identification of genets

Out of 592 samples, the genotyping of 11 microsatellite loci was successfully achieved in 586 samples (99%) (Table 2). The genotype accumulation curves indicated that the microsatellite loci from one to five could identify all multilocus genotypes present in each quadrat (Appendix S8), demonstrating that the newly developed 11 microsatellite markers had sufficient power to identify genets (clones) for this study. The values of *P*_gen_ and *P*_sex_ were low (<0.0001), suggesting that the probability of obtaining identical clones by chance was very low. Therefore, the identification of genets and the estimation of clonal structures in this study can be considered reliable.

### Clonal structure in young forests

**Figure 2:**
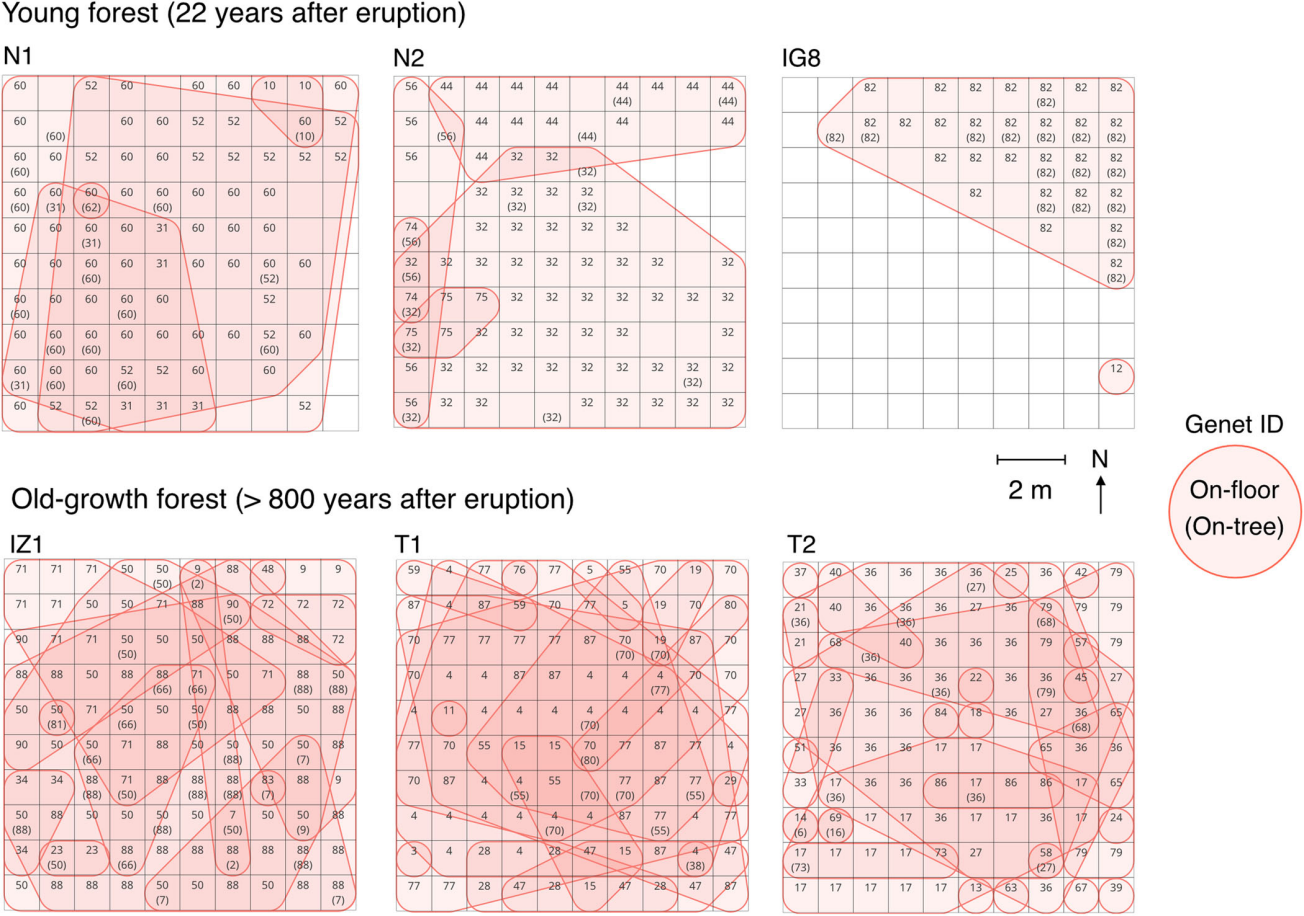
Distribution map of ramets and genets in the six 10 × 10 m quadrats. Each quadrat is divided into 1 m grids (*N* = 100). Numbers in the grids represent unique identifiers for genets. Numbers are shown in parentheses when ramets were at the on‐tree stage. Shapes of genets are denoted by solid polygons.

**Figure 3:**
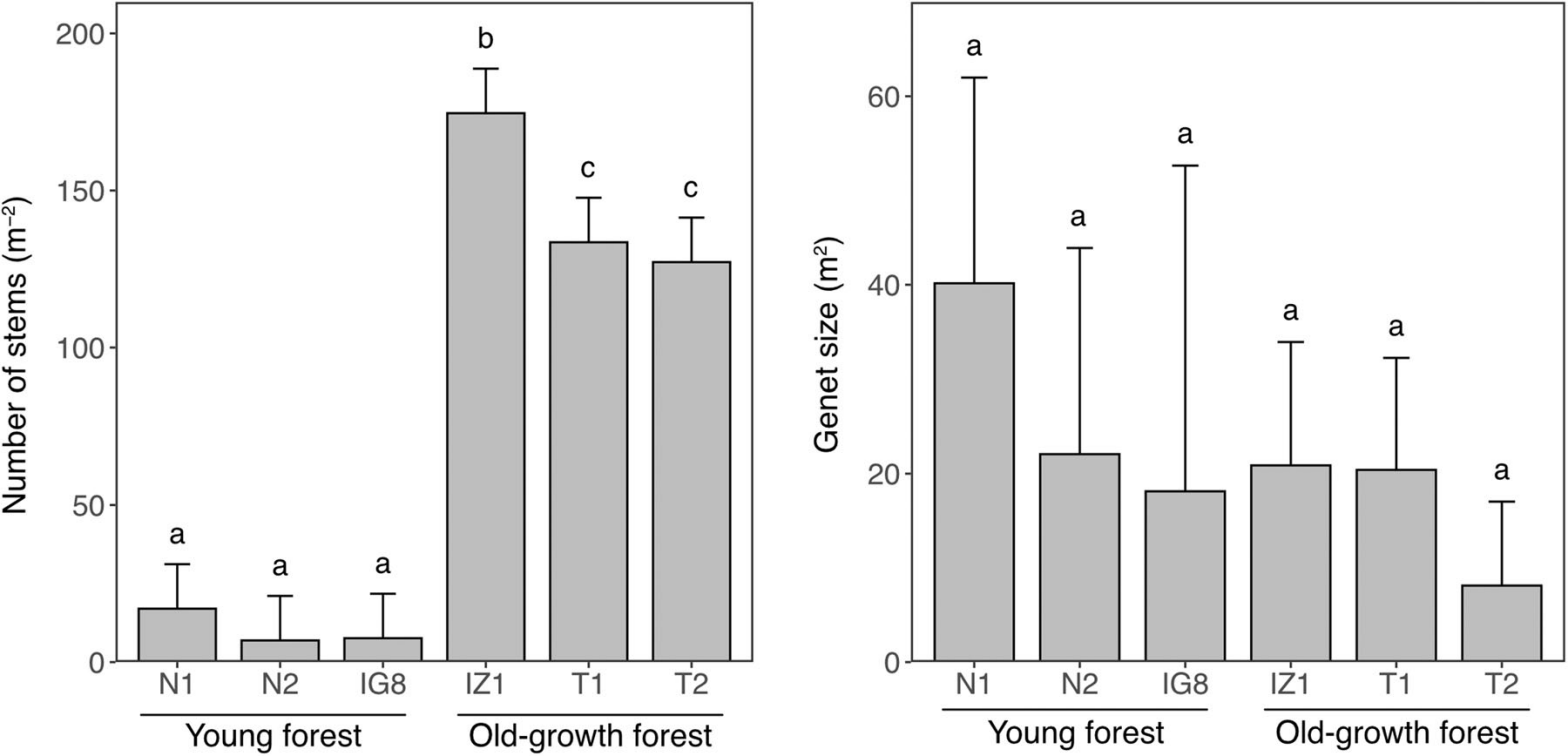
Numbers of stems (left panel) and sizes of genets (right panel) in the study sites. Mean values with the corresponding 95% confidence interval are shown for the six quadrats. Different letters indicate significant differences based on the Tukey comparison test (confidence level = 0.95).

**Figure 4:**
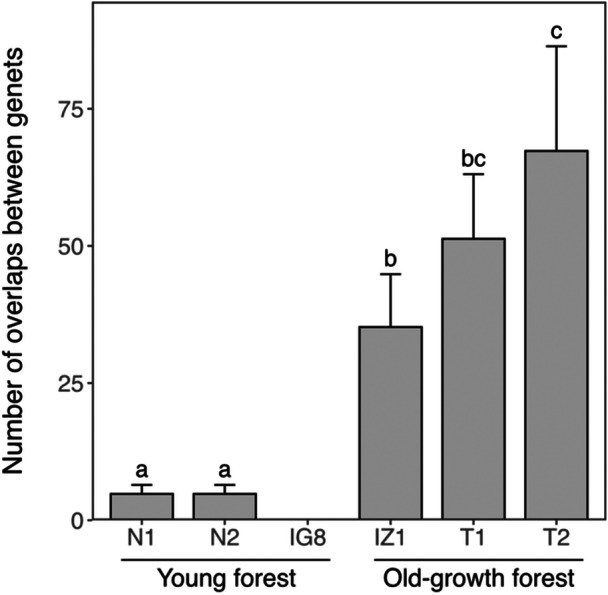
The number of overlaps between genets in each study site. Variability of the genet overlaps in the study sites was calculated by generating data sets that excluded one genet from all combinations. Error bars represent a 95% confidence interval for the generated data sets. Different letters indicate that 95% CIs did not overlap between the study sites.

In the young forest sites (N1, N2, IG8), a total of 231 ramets were identified, with an average of 77.0 ± 28.2 per quadrat (Table 1). These ramets comprised 12 genets, with an average of 4.0 ± 1.7 per quadrat (Figures 2 and 3). The percentage of clonal ramets (those constituting multiple‐ramet genets when a ramet was excluded from each genet) to the total number of ramets was 94.9 ± 0.6% on average (Table 2). These results indicate that the majority of ramets are derived from clonal reproduction, with few ramets originating from seed reproduction in the three young forest sites. At the on‐floor stage, at least one ramet was found in an average of 60.7 ± 27.52 grids per quadrat, while at the on‐tree stage, ramets were found in an average of 16.3 ± 2.5 grids per quadrat (Table 1), indicating that lianas were found approximately four times more frequently at the on‐floor stage than the on‐tree stage. The average number of ramets per square meter was 10.5 ± 10.4, with no significant differences among the young forest sites (Figure 3). The average size of genets was 28.9 ± 32.0 m² (Figure 3; Appendix S9), with no significant differences among the three young forest sites (Figure 3). There were also no significant differences in the number of overlaps between genets among the three young forest sites (Figure 4). These results indicate that the patterns of stem density, genet size, and genet overlaps are similar and consistent across the young forest sites.

**Table 1:** Numbers of ramets sampled and genotyped in this study. The ramets that were climbing trees (“on‐tree”) and those that had not yet started climbing (“on‐floor”) were randomly sampled, one per 1 × 1 m grid (*N* = 100) of six quadrats.

Forest type	Quadrat	Ramets (*n*)		
		On‐tree	On‐floor	Total
Young forest	N1	19	79	98
	N2	14	74	88
	IG8	16	29	45
	Mean ± SD	16.3 ± 2.5	60.7 ± 27.5	77.0 ± 28.2
Old‐growth forest	IZ1	29	100	129
	T1	12	100	112
	T2	17	97	114
	Mean ± SD	19.3 ± 8.7	99.0 ± 1.7	118.3 ± 9.3

**Table 2:** Summary of numbers of genets and ramets identified in this study.

Forest type	Quadrat	Genets (*n*)	Multi‐ramet genets (*n*)	Maximum number of ramets within a genet	Clonal ramets (%)^a^
Young forest	N1	5	4	68	94.9
	N2	5	5	57	94.3
	IG8	2	1	44	95.6
	Mean ± SD	4.0 ± 1.7	3.3 ± 2.1	56.3 ± 12.0	94.9 ± 0.6
Old‐growth forest	IZ1	14	11	44	89.2
	T1	17	12	28	84.8
	T2	30	11	38	73.7
	Mean ± SD	20.3 ± 8.5	11.3 ± 0.6	36.7 ± 8.1	82.6 ± 8.0

^a^ Percentage of ramets that constitute multiple‐ramet genets, calculated as 1 − total number of genets/total number of ramets.

### Clonal structure in old‐growth forests

In the old‐growth forest sites (IZ1, T1, T2), a total of 356 ramets were identified, with an average of 118.3 ± 9.3 per quadrat (Table 1). These ramets comprised 61 genets, with an average of 20.3 ± 8.5 per quadrat (Figures 2 and 3). The percentage of clonal ramets to the total number of ramets was 82.6 ± 8.0% on average (Table 2), which was lower than that in young forests but not significantly different (Fisher's exact test, *P* = 0.30, odds ratio = 1.14, 95% CI: 0.89–1.47). These results indicate that, while a large proportion of ramets are derived from the clonal reproduction in the three old‐growth forest sites, this proportion tended to be lower than in young forests. At the on‐floor stage, at least one ramet was found in an average of 99.0 ± 1.7 grids per quadrat, while at the on‐tree stage, ramets were found in an average of 19.3 ± 8.7 grids per quadrat (Table 1). This indicated that lianas were found approximately five times more frequently at the on‐floor stage than the on‐tree stage. The average number of ramets per square meter was 145.1 ± 36.1, with significant differences found between IZ1 and the other two sites (Figure 3). The average size of genets was 14.5 ± 23.2 m² (Figure 3; Appendix S9), with no significant differences among the three sites (Figure 3). There was a significant difference in the number of overlaps between IZ1 and T2 (Figure 4). These results indicate that while overall tendencies in genet size were similar, there were differences in stem density and in the frequency of genet overlaps within old‐growth forests.

### Comparison of clonality between young and old‐growth forests

The clonal diversity indices (*R*, *V*, *β*) were consistently lower in the young forest sites than in the old‐growth forest sites (Table 3). This suggests that the clonality of the study species in the old‐growth forests is characterized by greater richness and greater evenness. The number of ramets (stem density) was significantly higher in all old‐growth forest sites than in the young forest sites (Figure 3; Wilcoxon rank‐sum test, *P* < 0.001; Appendix S9). Specifically, the stem density in the old‐growth forests was >14 times greater than in the young forests, indicating that the clonal structure in old‐growth forests is characterized not only by greater clonal diversity, but also by greater abundance compared to young forests.

**Table 3:** Summary of clonal diversity indices: clonal richness (*R*), Simpson's evenness index (*V*), and Pareto index. Number in parentheses represents *P* values. Pareto index for IG8 was not calculated due to the very few number of genets (*N* = 2).

Forest type	Quadrat	*R*	*V*	Pareto
Young forest	N1	0.041	0.552	0.08 (0.04)
	N2	0.046	0.624	0.13 (0.01)
	IG8	0.023	0.000	–
Old‐growth forest	IZ1	0.102	0.785	0.22 (0.01)
	T1	0.144	0.878	0.33 (0.01)
	T2	0.257	0.754	0.27 (<0.001)

There were no significant differences in the size of the genets between the two forest types (Figure 3; Wilcoxon rank‐sum test, *P* = 0.46; Appendix S9). However, the numbers of overlapping genets were generally higher in the old‐growth forests than in the young forests (Figure 4). These results indicate that, while the genet size due to extensive clonal reproduction was similar between the sites, the clonal structure in the old‐growth forests was characterized by more overlaps of genets than in the young forests. The higher number of overlaps of genets in the old‐growth forests was further supported by the fraction of pairs of ramets sharing the same genet, which was consistently higher in young forests (0.66 ± 0.23) than in old‐growth forests (0.18 ± 0.07) up to a spatial distance of 7 m (Appendix S10).

## DISCUSSION

This study evaluated the clonal proliferation processes of the liana species *T. asiaticum* var. *asiaticum* following a large natural disturbance by comparing populations in young forests established soon after a volcanic eruption with those in old‐growth forests unaffected by the eruption. Our findings suggest that, following the eruption, a small number of genets were initially established via seed dispersal from surrounding areas, and that subsequent rapid colonization occurred primarily through extensive clonal reproduction by these genets. In addition, clonal reproduction also played a key role in long‐term increases in abundance and complexity in genetic structure. Contrary to our initial expectation, the relatively high genetic diversity and small genet sizes observed in old‐growth forests suggest that recurrent seedling recruitment, along with clonal growth from adjacent sites, likely contributed to maintaining and even enhancing genetic diversity over the long term. Thus, multiple reproductive processes, including both seed and clonal reproduction, appear to underpin the observed patterns of genetic diversity and spatial genetic structure in mature forest environments.

### Rapid colonization with clonal proliferation after large natural disturbance

It was confirmed that nearly all individuals (ramets) were derived from clonal reproduction, averaging 94.9% in young forests. This finding suggests that the colonization of lianas in young forests is driven predominantly by clonal reproduction. Previous studies have reported that lianas rapidly colonize following large‐scale natural disturbances such as typhoons and wildfires (Allen et al., 2005; Leicht‐Young et al., 2013); however, these disturbances do not eradicate regenerative plant propagules. Therefore, the results of those studies reflect the combined effects of surviving liana individuals, seed reproduction (seed dispersal, seed bank), and clonal reproduction, making it challenging to quantitatively assess the impact of clonal reproduction on liana proliferation. By contrast, the young forests in this study were established after the volcanic eruption that destroyed the vegetation (Yamanishi et al., 2003; Takahashi et al., 2008). Hence, the apparent increase in individual numbers through clonal growth observed here reflects the colonization by new individuals (seed‐originated) that dispersed from the surrounding environment and then proliferated through clonal growth, rather than from preexisting seeds and seedlings before the disturbance. The significant increase in lianas due to clonal reproduction in young forests suggests that the rapid proliferation following large disturbances reported in other forest communities (e.g., Allen et al., 1997; Barry et al., 2015) could also largely be attributed to clonal reproduction. This finding underscores the importance of considering clonality when understanding liana proliferation after disturbances.

The low number of genets found in the young forest sites (an average of four genets per quadrat) suggests that the study species increased its abundance through clonal reproduction from a few seed‐originated individuals. In addition, given the absence of the species in these sites 10 yr prior to the sampling for this study, these individuals likely dispersed and established within that decade (Appendix S3). The extensive vegetative decline caused by the eruption necessitates seed dispersal from the surrounding environment for establishment in young forest sites (Antos and Zobel, 1986; Wood and del Moral, 1987; Nishi and Tsuyuzaki, 2004). However, despite this necessity, the contribution of seed reproduction to the overall increase in abundance after new colonization in young forests was relatively small. Although wind dispersal may have resulted in a vast number of seeds being dispersed even before forest formation, the species appears to have established only after the forest was formed after the eruption (Appendix S3). This suggests that the harsh environmental conditions prior to forest formation may have hindered seedling establishment via seed reproduction. The study species is known to proliferate along the edges of evergreen forests in Japan (Yamazaki, 1993), and its seedlings exhibit pronounced elongation growth under relatively bright conditions (Kato et al., 2012). Nevertheless, the relatively low number of seed‐originated individuals may be attributable to limited seed sources in the surrounding area and/or other environmental factors such as soil nutrient limitations (Eriksson and Ehrlén, 1992; López et al., 2010; Peng et al., 2021). To further clarify these factors, it is necessary to elucidate the life history strategy of seed reproduction by long‐term monitoring of the survival and environmental response of lianas at the stages from seed to seedling (Mori et al., 2020).

### Long‐term increase in abundance and genetic complexity by clonal reproduction

The large number of individuals observed in the old‐growth forest sites indicates that the liana species had increased its stem density through long‐term clonal reproduction, forming a complex genetic structure. Specifically, stem density per square meter in the old‐growth forests was 14 times greater than in the young forests, highlighting the significance of clonal reproduction in the long‐term increase in liana abundance in closed canopy forests. The contribution of clonal reproduction to the increase in liana abundance was consistent with previous findings (Yorke et al., 2013; Schnitzer et al., 2021). In the tropical old‐growth forests of Costa Rica, a study reported that over an 8 yr period, the number and basal diameter of lianas increased by 15%–20%, part of this increase being attributed to clonal growth (Yorke et al., 2013). Additionally, in the tropical forests of Barro Colorado Island, Panama, the number of liana stems increased by ~30% over 10 yr following small‐scale natural disturbances, and many of these increases were attributed to clonal coppiced stems (Schnitzer et al., 2021). This indicates that clonal reproduction plays a central role in the long‐term proliferation of lianas in various forest communities.

The significantly larger abundance found in the old‐growth forests compared to young forests in this study could have been due to the extensive overlapping (intermingling) of genets derived from clonal reproduction. The high level of intermingling of genets indicates that the study species exhibits the “guerilla” type growth form (Schmid and Harper 1985). Clonal plants are known to form high‐density and complex spatial genetic structures through the intermingling of genets with extensive clonal growth (e.g., Kitamura et al., 2001; Reisch et al., 2007; Ohsako, 2010). This characteristic of clonal growth could have decreased the cost of clonal reproduction and increased reproductive fitness, as reported in a dwarf bamboo (Matsuo et al., 2014). In the old‐growth forest sites, the dark light environment and high ramet density on the forest floor likely made seedling establishment through seed reproduction difficult. However, more genets (five times as many, on average) were identified in the old‐growth forest sites than in the young forest sites. These findings suggest that both continuous new recruitment through seed reproduction and an increase in stem density through clonal reproduction had shaped the spatial genetic structure and abundance of the liana species in the old‐growth forests. Indeed, large‐scale disturbances have been shown to significantly alter the abundance and genetic structure and diversity in forest plants (Jackson and Fahrig, 2014; Davies et al., 2016; Soares et al., 2019). Furthermore, prolonged clonal growth of herbaceous plant populations has been recognized as a key factor enabling long‐term persistence following such disturbances, including habitat fragmentation in forests (Honnay et al., 2005). These findings indicate that recruitment through seed reproduction, combined with extensive clonal reproduction, shapes the genetic structure and diversity of liana populations, with clonal reproduction playing a more dominant role in long‐term liana proliferation following large disturbances.

To further contextualize our findings, we compared our study species with dwarf bamboo, a well‐studied, long‐lived (>60 yr; Campbell, 1985) clonal plant that reestablishes exclusively from newly dispersed seeds after synchronized flowering events. Previous studies have shown that, following a synchronized flowering event, dwarf bamboo (e.g., *Sasa kurilensism* and *S. tsuboiana*) exhibits peak clonal diversity afterward due to high seed production and a large number of seedlings (Makita, 1992; Makita et al., 1993). The number of seedlings then declines over the following decade at a rate of ~0.18/yr (Makita, 1992). Kitamura and Kawahara (2009) demonstrated that, in a 10 × 50 m plot at the time of flowering, only six genets were detected among 1267 clumps (comprising 2529 living culms), with 1176 of these clumps belonging to a single clone—indicating markedly low clonal diversity before flowering in *Sasa cernua*. Thus, in dwarf bamboo, seed reproduction is critical during the establishment phase, but as time progresses, its reliance on synchronized flowering leads to an overall reduction in clonal diversity. By contrast, while both our study species and dwarf bamboo initially depend on seed reproduction for establishment, *T. asiaticum* var. *asiaticum* not only rapidly colonizes disturbed sites through clonal expansion, but also maintains and even enhances clonal diversity over time—particularly in old‐growth forests—through continuous recruitment via seed dispersal as well as clonal growth by external clones. This contrast reflects differences in initial recruitment patterns and subsequent reproductive strategies. In our study system, initial recruitment was likely limited by low seed availability following the volcanic eruption, and clonal diversity gradually increased as seed inflow continued. Continuous local reproduction through repeated flowering may have contributed to both the recruitment of new genets and the maintenance of high genetic diversity in old‐growth forests. This comparison underscores the distinctive temporal dynamics of clonal reproduction among species, which arise from both species‐specific life history traits and environmental conditions following disturbance. These contrasting trajectories suggest that clonal diversity is likely to increase during succession when continued seed input and favorable environmental conditions permit the recruitment of new genets, but to decrease where such recruitment is limited and clonal expansion of a few founders dominates.

## CONCLUSIONS

Our results demonstrate that clonal growth is crucial for the rapid and long‐term increase in abundance (stem density)—and that both seed and clonal reproduction enhance the genetic diversity (genetic structure and clonal diversity)—of the liana species after large‐scale natural disturbances. The processes suggested by this study are as follows: (1) After the volcanic eruption, a few genets that had established via seed dispersal into the recovering *Alnus* shrublands increased their abundance through extensive clonal growth on the forest floor. (2) Over time, as the forests transitioned into climax communities dominated by old‐growth *C. sieboldii*, clonal growth significantly increased the abundance of the liana species, resulting in high‐density ramets and complex spatial genetic structures. Meanwhile, repeated seedling establishment and clonal expansion from adjacent sites further contributed to greater clonal diversity.

Thus, *T. asiaticum* var. *asiaticum* exhibits both pioneer characteristics and high shade tolerance. It rapidly establishes in bright shrublands through both seed and clonal reproduction, while achieving long‐term increases in abundance through clonal growth and further enhancing genetic diversity via continuous seed and clonal reproduction in closed‐canopy old‐growth forests. These findings indicate that the combination of clonal and seed reproduction enables the liana species to adapt effectively to forest succession and environmental changes following large‐scale disturbances.

Increasing liana dominance has primarily been reported in tropical forests (Ngute et al., 2024), partly due to their high diversity and abundance in these regions. However, our results suggest that clonal reproduction can significantly enhance liana proliferation after large‐scale disturbances in higher latitudes, such as temperate regions. As large‐scale disturbances increase worldwide (Siedl et al., 2017), lianas may continue to proliferate across various forest types. Given their structural and functional importance in forest ecosystems and significant impact on community dynamics, lianas are increasingly considered in future predictions of forest communities (van der Heijden et al., 2013; Meunier et al., 2021). This study underscores the necessity of evaluating the clonal nature of lianas to comprehensively understand and predict changes in their abundance and genetic diversity caused by forest disturbances.

## AUTHOR CONTRIBUTIONS

H.M. and T.K. conceived the study design and conducted the field survey. H.M. performed the molecular experiments, analyzed the data, and wrote the manuscript, with T.K. providing editorial advice.

## Supporting information


**Appendix S1.** Photographs of Miyake‐jima Island after the 2000 eruption and the study species in the two forest types at the study sites in 2024.

**Appendix S2.** Stages of primary succession and vegetation changes over time in Miyake‐jima Island.

**Appendix S3.** Change in abundance of the study species on the forest floor in the six quadrats in 2012 and 2020.

**Appendix S4**. Stratified vegetation cover (%) in the study sites.

**Appendix S5**. Volcanic ash deposition of the study sites.

**Appendix S6.** Characteristics of 11 microsatellite loci for *Trachelospermum asiaticum* var. *asiaticum*.

**Appendix S7.** Methods for the development of 11 microsatellite markers.

**Appendix S8.** Genotype accumulation curves of the 11 SSR markers developed in this study. Horizontal dashed line indicates the maximum number of multilocus genotypes identified in the study site (MLG).

**Appendix S9.** Genet size and stem counts in young and old‐growth forest sites.

**Appendix S10.** Spatial distance and the corresponding probabilities of clonal identity.

## Data Availability

The data that support the findings of this study are available in Zenodo at https://doi.org/10.5281/zenodo.15679451.
