# Supplementary material for: Clonal reproduction as a driver of liana proliferation following large‐scale disturbances in temperate forests
Source: Am J Bot. 2025 Aug 13;112(8):e70085. doi: 10.1002/ajb2.70085 (PMC12374572; doi:10.1002/ajb2.70085)

Appendix S1. Photographs of (a) Miyake-jima Island after the 2000 eruption and (b) the study species in the two forest types at the study sites in 2024.

(a) Miyake-jima island after the 2000 eruption

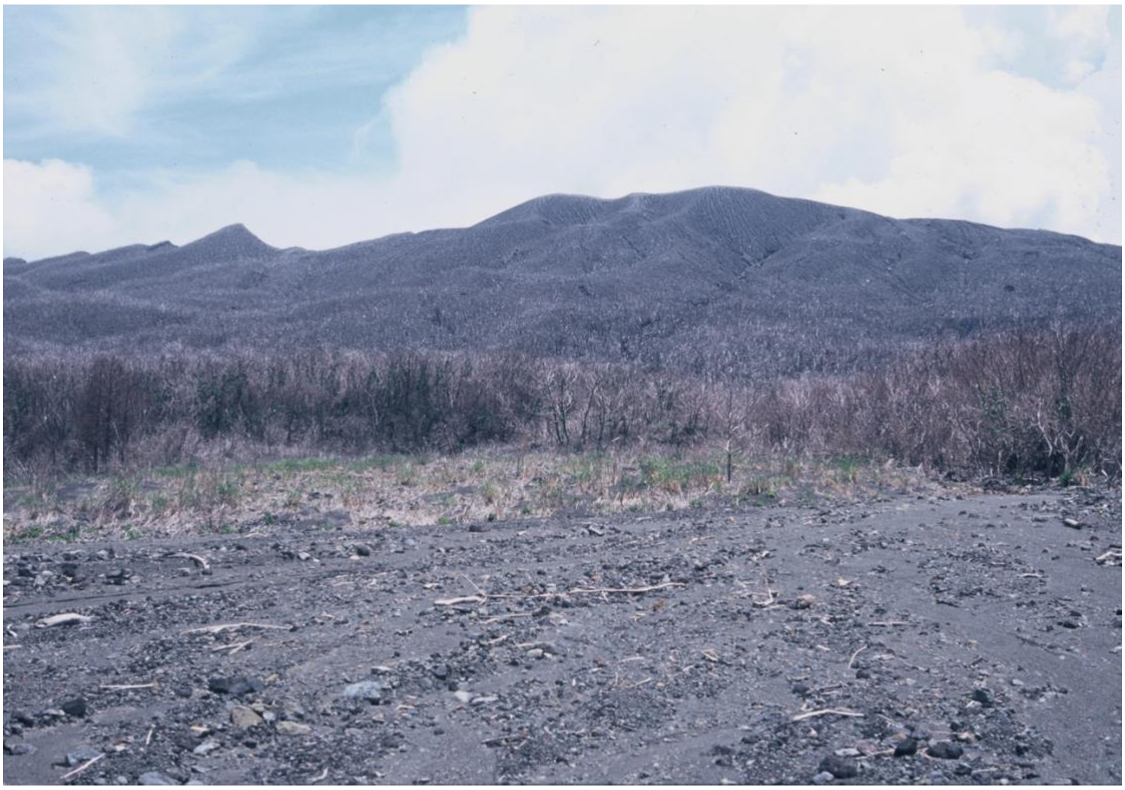

Appendix S1. *continued*

(b)

Young forest

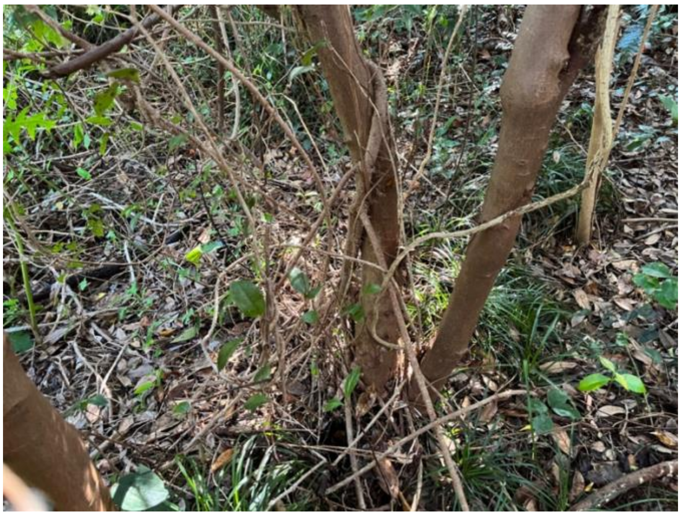

Old-growth forest

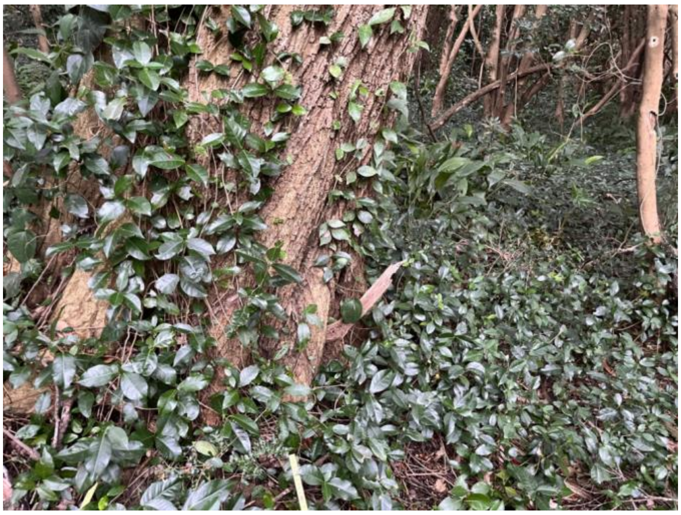

Supplement: Supplementary file 1 — Appendix S1. Photographs of Miyake‐jima Island after the 2000 eruption and the study species in the two forest types at the study sites in 2024. [file AJB2-112-e70085-s005.pdf]
