# Supplementary material for: Clonal reproduction as a driver of liana proliferation following large‐scale disturbances in temperate forests
Source: Am J Bot. 2025 Aug 13;112(8):e70085. doi: 10.1002/ajb2.70085 (PMC12374572; doi:10.1002/ajb2.70085)
Supplement: Supplementary file 2 — Appendix S2. Stages of primary succession and vegetation changes over time in Miyake‐jima Island. [file AJB2-112-e70085-s004.pdf]

Appendix S2. Stages of primary succession and vegetation changes over time in Miyakejima Island.

Table was created based on Kamijo et al. (2002).

**Stage (yr-old)**	**Primary Succession Process**
0	Bare land
16	Colonization of *Alnus* and *Reynoutria*
37	*Alnus* shrub
-	Facilitation by N-fixation of *Alnus*.<br>Colonization of *Prunus* and *Machilus*.<br>Rapid above-ground-biomass accumulation.
125	*Machilus* and *Prunus* forest
-	Disappearance of *Alnus* and *Prunus*.<br>Colonization of *Castanopsis*.
> 800	*Castanopsis* forest
