# Supplementary material for: Clonal reproduction as a driver of liana proliferation following large‐scale disturbances in temperate forests
Source: Am J Bot. 2025 Aug 13;112(8):e70085. doi: 10.1002/ajb2.70085 (PMC12374572; doi:10.1002/ajb2.70085)
Supplement: Supplementary file 3 — Appendix S3. Change in abundance of the study species on the forest floor in the six quadrats in 2012 and 2020. [file AJB2-112-e70085-s001.pdf]

Appendix S3. Change in abundance of the study species on the forest floor in the six quadrats in 2012 and 2020 based on Braun-Blanquet cover-abundance scale.

Year	Young forest			Old-growth forest		
	IG8	N1	N2	T1	T2	IZ1
2012	+			44	44	44
2020	11	22	22	44	44	44
