# Supplementary material for: Clonal reproduction as a driver of liana proliferation following large‐scale disturbances in temperate forests
Source: Am J Bot. 2025 Aug 13;112(8):e70085. doi: 10.1002/ajb2.70085 (PMC12374572; doi:10.1002/ajb2.70085)
Supplement: Supplementary file 4 — Appendix S4. Stratified vegetation cover (%) in the study sites. [file AJB2-112-e70085-s006.pdf]

Appendix S4. Stratified vegetation cover (%) in the study sites. Numbers in the parentheses represent the height (m) of the stratum. The surveyed was conducted in 2018.

	Young forests			Old-growth forests		
Stratum	IG8	N2	N1	T1	T2	IZ1
Canopy Stratum	0	0	0	70 (10–13)	90 (10–14)	90 (10–15)
Subcanopy Stratum	40 (3–8)	90 (3–8.5)	90 (3–8)	10 (3–10)	50 (4–10)	10 (5–8)
Shrub Stratum	100 (1–3)	20 (1–3)	60 (1–3)	30 (0.7–3)	10 (1–4)	50 (1–5)
Herbaceous Stratum	15 (0–1)	20 (0–1)	30 (0–1)	50 (0–0.7)	80 (0–1)	70 (0–1)
