# Supplementary material for: Clonal reproduction as a driver of liana proliferation following large‐scale disturbances in temperate forests
Source: Am J Bot. 2025 Aug 13;112(8):e70085. doi: 10.1002/ajb2.70085 (PMC12374572; doi:10.1002/ajb2.70085)
Supplement: Supplementary file 5 — Appendix S5. Volcanic ash deposition of the study sites. [file AJB2-112-e70085-s008.pdf]

Appendix S5. Volcanic ash deposition of the study sites. The survey was conducted in 2012.

Forest types	Quadrats	Volcanic ash deposition (cm)
Young forests	N1	23
	IG8	38
Old-growth forests	IZ1	0
	T1	0
