# Supplementary material for: Clonal reproduction as a driver of liana proliferation following large‐scale disturbances in temperate forests
Source: Am J Bot. 2025 Aug 13;112(8):e70085. doi: 10.1002/ajb2.70085 (PMC12374572; doi:10.1002/ajb2.70085)
Supplement: Supplementary file 6 — Appendix S6. Characteristics of 11 microsatellite loci for Trachelospermum asiaticum var. asiaticum. [file AJB2-112-e70085-s003.pdf]

Appendix S6. Characteristics of 11 microsatellite loci for *Trachelospermum asiaticum* var. *asiaticum.*

ID	Forward primer sequence	Reverse primer sequence	SSR motif sequence	PCR Product size range (bp)	*Na*	*Ne*	*Ho*	*He*
Ta01	GCCTTGCCAGCCCGCAAAGTAG GGAGAGGAGGGAGTGGC	GTTTCTTACCAACACTT CATTTCATCCAAGGC	(AG)13	95-145	6	3.760	0.500	0.734
Ta02	CAGGACCAGGCTACCGTGACTG AGTGGAATACAGGAGGTCTTC	GTTTCTTGCTCGTAAAC CCTGCAACCCAAC	(CT)13	135-170	4	1.907	0.448	0.476
Ta03	CGGAGAGCCGAGAGGTGTGGT GGGATGATGTAGTGGGTGG	GTTTCTTAAACTAAGCA CCACCAACCGCGC	(TC)18	160-250	21	14.885	0.897	0.933
Ta04	GCCTTGCCAGCCCGCAGTCTTG CCTCTTCAGAATCTGGTG	GTTTCTTGTCATTCCAC AAGAGAGACTGGTC	(TC)25	90-150	8	5.455	0.900	0.817
Ta05	GCCTTGCCAGCCCGCTTCAGGT TGTGAAGTGTGGCATC	GTTTCTTGCTCAAGTGC TCCAGGACAAGAC	(TA)14	140-200	4	2.985	0.700	0.665
Ta06	CAGGACCAGGCTACCGTGCTCG AGTATCATTCATTTGGCAAGG	GTTTCTTAAATCAGTTA CGACAGAGGGTGC	(TA)13	180-240	3	1.474	0.241	0.322
Ta07	CGGAGAGCCGAGAGGTGCCTG GATCTTTGCATTTGAACTTGCC	GTTTCTTAAATGATAGA GAGTAGCACACTGTCC	(AG)13	130-170	7	4.865	0.733	0.794
Ta08	GCCTCCCTCGCGCCATCCAAAT CACCAGTTCACCACACAG	GTTTCTTGCATGTTGTTA CTGTATGTTGGC	(AT)28	90-110	6	2.885	0.667	0.653
Ta09	GCCTTGCCAGCCCGCAACGAAT CACCGTGACTGGCAG	GTTTCTTGCATCGCTTG GCATTTGTGC	(AT)19	157-190	9	6.294	0.733	0.841
Ta10	GCCTTGCCAGCCCGCTCGCTCA CCTTAGCTTAGTAAATCAC	GTTTCTTCTCCATCTCTC ACTCTCGGCTAC	(GA)23	166-196	11	2.711	0.533	0.631
Ta11	GCCTTGCCAGCCCGCACACGTA TGATTTGACCCATGCCC	GTTTCTTCATGCATACG TAAGCTAAATTCCGG	(TA)14	125-150	4	2.278	0.800	0.561
