# Supplementary material for: Clonal reproduction as a driver of liana proliferation following large‐scale disturbances in temperate forests
Source: Am J Bot. 2025 Aug 13;112(8):e70085. doi: 10.1002/ajb2.70085 (PMC12374572; doi:10.1002/ajb2.70085)

Appendix S8. Genotype accumulation curves of the 11 SSR markers developed in this study. Horizontal dashed line indicates the maximum number of multilocus genotypes identified in the study site (MLG).

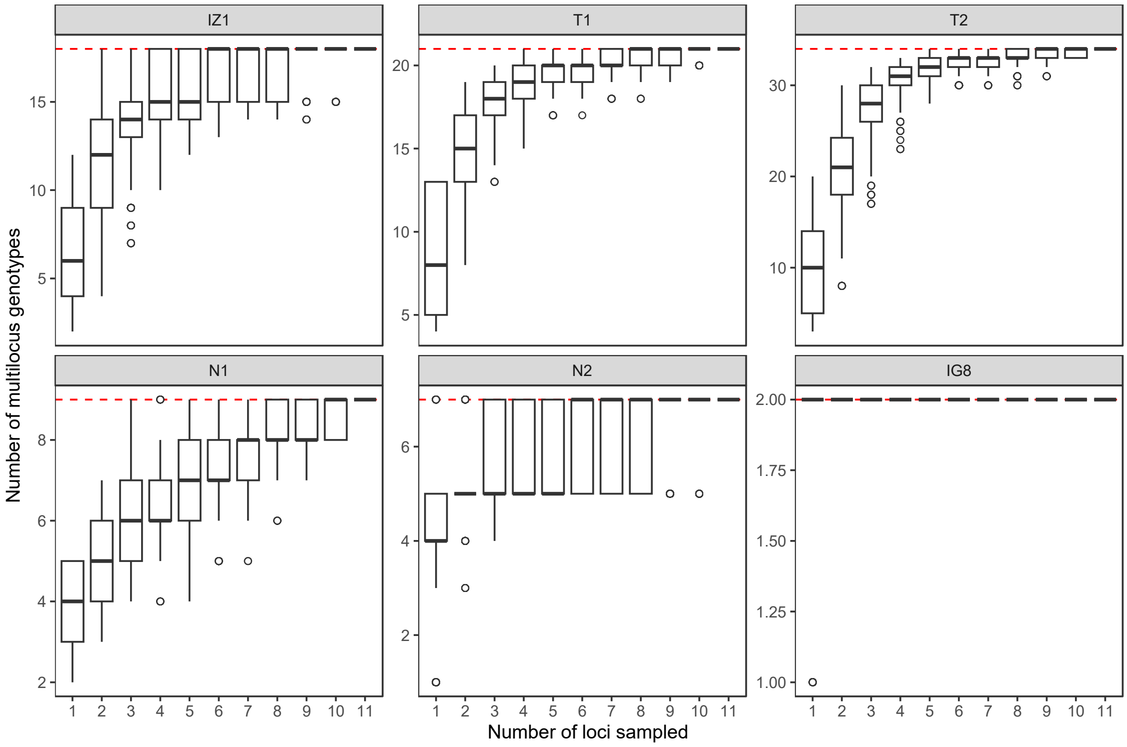

Supplement: Supplementary file 8 — Appendix S8. Genotype accumulation curves of the 11 SSR markers developed in this study. Horizontal dashed line indicates the maximum number of multilocus genotypes identified in the study site (MLG). [file AJB2-112-e70085-s009.pdf]
