# Supplementary figures and images for: Clonal reproduction as a driver of liana proliferation following large‐scale disturbances in temperate forests
Source: Am J Bot. 2025 Aug 13;112(8):e70085. doi: 10.1002/ajb2.70085 (PMC12374572; doi:10.1002/ajb2.70085)

**Appendix S9.** Genet size and stem counts in young and old-growth forest sites.

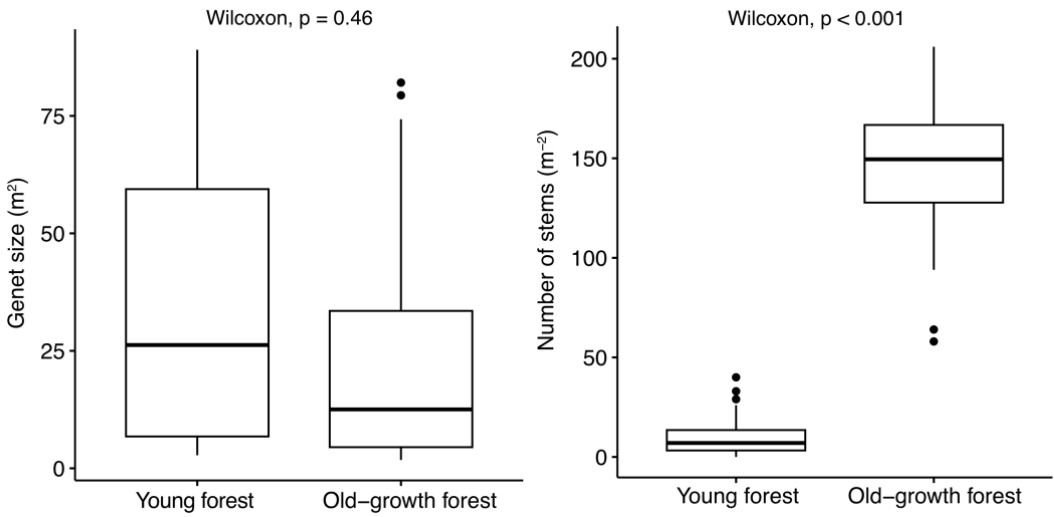

Supplement: Supplementary file 9 — Appendix S9. Genet size and stem counts in young and old‐growth forest sites. [file AJB2-112-e70085-s007.pdf]
