# Supplementary material for: Clonal reproduction as a driver of liana proliferation following large‐scale disturbances in temperate forests
Source: Am J Bot. 2025 Aug 13;112(8):e70085. doi: 10.1002/ajb2.70085 (PMC12374572; doi:10.1002/ajb2.70085)

Appendix S10. Spatial distance and the corresponding probabilities of clonal identity (the fraction of pairs of ramets sharing the same genet; Fr) in the study sites.

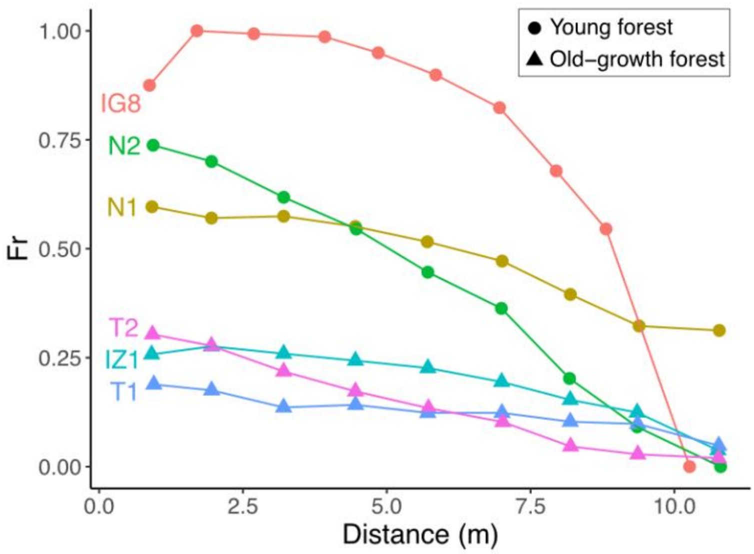

Supplement: Supplementary file 10 — Appendix S10. Spatial distance and the corresponding probabilities of clonal identity. [file AJB2-112-e70085-s002.pdf]
